# Address, Read before the Merrimack Valley Dental Association

**Published:** 1865-12

**Authors:** A. Lawrence


### ADDRESS.

[Read before the Merrimack Valley Dental Association, Nov. 2d, 1865.]

BY A. LAWRENCE.

Gentlemen: — We meet to-day in this proud capital of the Old Granite State to lay on the altar of our Association renewed pledges of professional fealty and respect.

Here on the banks of the tranquil stream, whose name we take, where from its source to the Atlantic, the busy hum of the spindle, the clanking loom, and the sonorous anvil have superceded the war-hoop, tomakawk, and scalping knife, we welcome each other to the trials, duties, and pleasures, incident to our annual meeting.

Here we challenge free discussion, free thought and opinion upon whatever pertains to our professional advancement, that we may render ourselves respectable, and be respected—useful, and be appreciated.

Before proceeding to the consideration of the more important and interesting matters which will shortly claim your attention, I propose by your indulgence, to present a few thoughts, engendered by the circumstances connected with the formation, and present status of our Association.

Our organization was consummated two years ago, this day, while our country was engaged in subduing a licentious, and rebel horde, to the mild and wholesome laws, whose requirements they had set at defiance.

New armies were being raised, and old ones, thinned out, by traitorous steel, starvation and disease, were being replenished from the patriotic thousands who hold their country's integrity of more value than mere party agrandizement.

Every one was interested in the war—the loyal in the success of our arms; the disloyal, in discouraging enlistments, and in their blatant vituperations against everything the government either did, or did not do, to favor that supineness and treachery so essential to the success of the rebellion.

For the time, filling quotas and substituting men, took the place of filling and substituting teeth, i. e. among dentists.— While we were less concerned how to obtain gold, even at 299, than how to rid ourselves of the superabundance of *copper* by which we were surrounded at any price.

The time was inauspicious, but the perseverance of those most active in the work, accomplished that, which in their judgement, would conduce to the prosperity of the profession; and the Merrimack Valley Dental Association stood forth as another small star, in the grand constellation which lights up our professional firmament.

May this our "star in the east" guide us to greater usefulness, and continue to illumine the pathway that leads to the temple of knowledge, long after we shall have passed through the portals made sweet by the skirts of our blessed Redeemer.

We may be allowed the indulgence, in a reasonable degree of satisfaction, in view of our success, thus far, and of our standing in the circle of similar associate bodies throughout the country.

The National Association, lately in session at Chicago, has satisfactorily recognized us — there being no desire manifested to "leave us out in the cold;" on the contrary, we were welcomed into that body of warm hearted and intelligent men, as brothers and co-workers in an enobling and useful profession.

I may be permitted to say in my own behalf, that I was amply repaid for the time, and expense of attendance, in the pleasure of listening to those more learned and experienced than myself; I have returned with a new lease of life, and a greater determination to be useful to the extent of my poor abilities, that when called hence, it may not be said of me, he left no foot-prints to mark his going forth. It is invigorating to draw in the exalted atmosphere of enlightened presence.

It mollifies the soul, and roots out the weeds of envious discord — heals the wounded spirit of conscious integrity, and covers with the mantle of charity, the digressions and short-comings of our fellows. Then let our national gatherings find full representation from all our local societies. Let this association at least, not fail to send its full quota, though small.

For the want of material, geographical position forbidding, we cannot reasonably anticipate a numerous membership; yet, that simple fact presents no barrier to our associated, or individual success; and considering the short time since our organization, we ought to be abundantly satisfied with the endorsement thus far bestowed. The Merrimack Valley contains but thirty or forty dentists, all told, of which numbers fourteen were present at the organization, and became members thereof. Twelve others have since joined, and one of the original number, Dr. Vinall, has died, thus showing that we have twenty-five "good men and true" enrolled in the work of regenerating and exalting the status of our profession in the Merrimack Valley. In what other section of the country have a full majority come up so manfully to endorse and aid in an organized effort to give character to their calling?

Now, having established our Association upon the sure foundation of success, shall we rest satisfied with semi-annually going through the dull forms of legislation, inseperable from such bodies? No, gentlemen, let us thoroughly subsoil the entire field of our labor, and bring to light the rich treasures that lie beneath the surface, wherewith to store our minds and add something to the general stock of useful information.

One grand object we have in view, is that modes of practice may be investigated, compared, harmonized and improved, all in the light of sound theory. Take if you please, filling teeth with gold, a subject sufficiently discussed, it would seem, to be well understood, and successfully executed by every practitioner in the land, yet such is not the fact, for the matter is receiving more attention now, than ever before.

Perhaps the most marked innovation upon the time honored method of consolidating the gold in filling teeth, is the introduction of the mallet for that purpose, by Dr. W. H. Atkinson, of New York. That so novel a departure from ordinary practice should meet with indifference, opposition and ridicule from a part of the profession, and be hailed with satisfaction and delight by the rest, is not strange, it is simply human. In this matter opinions must certainly conflict, but if we act on the saying, " prove all things, and hold fast that which is good," we shall arrive at pretty correct conclusions respecting the merits of the implement in question.

My own experience with the mallet, although I was at first, not a little prejudiced against it, has thus far been favorable, and there is no doubt in my mind, that a filling of greater density can be made with, than without it; but manipulative skill is requisite.

The mallet will not answer as a substitute for brains.

As with the matter just referred to, so with the entire list of operations we are called upon to perform. There is a great diversity of practice, every one honestly thinking his own mode the preferable one, whether attended with the best success, or not. Again, if we sail over the jaundiced sea of theory, and angle with our thoughts for ideas, we shall find our prizes of as many shades of value, as are the finny tribes that sport beneath old ocean's tides, and in many cases will require the same treatment, to render them palatable. In angling for ideas, remember that the largest fish, the deepest swim.

In conclusion, I congratulate you upon the stand you have taken, and the prospect before you. Go on! Batter down the begrimed walls that incarcerate our prejudices, that they may no longer, like vultures prey upon our better natures, but take their flight to the festering fields of isolation and illiberality. Persevere in the good cause! Build up the temple of learning another story, and leave no vacant chambers there.

